# Reduced 20-Hydroxyecdysone Signaling Modulates Antifungal Immune Responses and Growth in *Lymantria dispar* Against *Beauveria bassiana*

**DOI:** 10.3390/insects17080839

**Published:** 2026-08-13

**Authors:** Jingyu Cao, Yue Zhang, Jingxin Cao, Jianyang Bai, Chuanwang Cao, Zhe Xu, Ling Ma

**Affiliations:** 1 Department of Forest Protection, College of Forestry, Northeast Forestry University, 26 Hexing Road, Harbin 150040, China; 2 College of Life Sciences, Northeast Forestry University, Harbin 150040, China; 3 School of Forestry & Landscape Architecture, Anhui Agricultural University, Hefei 230036, China

**Keywords:** growth–immunity, trade-off, 20-hydroxyecdysone, innate immune, *Beauveria bassiana*, *Lymantria dispar*

## Abstract

20-Hydroxyecdysone (20E) is the active form of the steroid hormone ecdysone and is responsible for regulating insect development, molting, and metamorphosis. Infection with the entomopathogenic *Beauveria bassiana* enhances innate immunity and reduces 20E titers in *Lymantria dispar*. To further explore the role of the 20E signaling pathway in antifungal immunity, we combined exogenous hormone assays, proteomic profiling, and RNAi-mediated knockdown. In this study, exogenous hormone assays revealed that 20E accelerated the death of *B. bassiana*-infected larvae, whereas juvenile hormone (JH) markedly delayed it. 20E promoted larval weight gain and food utilization efficiency in *L. dispar* during fungal infection compared to the *B. bassiana* group. Moreover, the 20E + *B. bassiana* group significantly decreased the expression of the Toll pathway-related genes, *Attacin2*, *Gloverin4*, and *Lysozyme4*, while reducing hemocyte counts and *Pvf2* expression. Furthermore, the 20E + *B. bassiana* group increased the number of hyphae in the hemolymph, but had no obvious effect on hemocyte encapsulation capacity compared to the *B. bassiana* group. Proteomic analysis showed that Cyp306a1 and Cyp314a1 were significantly down-regulated in *L. dispar* at 48 h post-infection with *B. bassiana*. Knockdown of *Cyp306a1* delayed molting and increased larval mortality in *L. dispar*, but did not alter the mortality rate upon *B. bassiana* infection compared to the dsGFP group. Intriguingly, dsCyp306a1 + *B. bassiana* group had significantly increased the expression of *Toll3*, *Toll5*, *Toll7*, *Gloverin4* and *Cecropin5* compared to the *B. bassiana* group. In conclusion, these findings suggest that reduced 20E signaling shifts *L. dispar* from growth toward immune activation during *B. bassiana* infection. This study provides valuable insights into the 20E-mediated trade-off between growth and immunity.

## Simple Summary

*Lymantria dispar* is one of the most destructive defoliating pests worldwide, feeding on over 300 species of coniferous and deciduous trees and posing a serious threat to forest productivity and ecological health. Biological control with entomopathogenic fungi, such as *Beauveria bassiana*, has attracted considerable interest and wide application in agricultural and forestry pest management. However, the robust innate immune system of *L. dispar* greatly limits the insecticidal efficacy of *B. bassiana*. 20-Hydroxyecdysone (20E) is an endogenous insect hormone, and previous studies have primarily focused on its regulatory roles in development, molting, and metamorphosis. In our preliminary experiments, we observed that *B. bassiana* infection significantly reduced the 20E titer, decreased larval weight, and simultaneously triggered innate immune responses in *L. dispar*. Accordingly, the present study aims to investigate the role of 20E in regulating innate immunity and to elucidate its modulatory effects on both growth and immune defense during *B. bassiana* infection.

## 1. Introduction

20-Hydroxyecdysone (20E) is the active form of the steroid hormone ecdysone in insects [1]. Its biosynthesis is governed by the Halloween gene family, which includes Cyp307a1, Cyp306a1, Cyp302a1, Cyp315a1, and Cyp314a1 [2,3]. Upon synthesis, 20E binds to the ecdysone receptor (EcR) and ultraspiracle (USP), forming the 20E/EcR/USP complex. This complex subsequently activates the Broad-Complex (Br-C), Ecdysone-induced protein 75 (Eip75 or E75), Eip74EF, Eip78, hormone receptors (HR3, HR4, HR38, HR96), and FTZ-F1, thereby playing a regulatory role in the processes of molting, metamorphosis, and development [4,5,6]. In *Nilaparvata lugens*, *Helicoverpa armigera*, and *Drosophila melanogaster*, the 20E signaling pathway regulates insect growth, development, and reproduction capacity [7,8,9]. Many studies have shown that 20E is also involved in the regulation of innate immunity in insects [10,11]. For example, in *H. armigera*, 20E significantly increased the expression of pattern recognition receptors (PRRs) and antimicrobial peptides (AMPs) [12]. In *Locusta migratoria*, 20E significantly increased the expression of peptidoglycan recognition proteins, AMPs, and lysozyme, thereby enhancing its immune function [13]. By contrast, in *Bombyx mori*, injection of 20E significantly decreased the expression of AMPs and reduced antibacterial activity [14]. In *H. armigera*, injection of 20E (500 ng/larva) promoted the release of GAPB from oocytes and suppressed plasmatocyte spreading [15].

Immune activation consumes massive resources, often leading to trade-offs between growth and development and immune defense in insects [16]. Consequently, immune responses are commonly suppressed during development and reproduction [17]. For example, in *Allonemobius socius*, host immune capacity is significantly reduced after mating [18], and mating success is positively correlated with the duration of immune suppression in *Tenebrio molitor* [19]. During the reproductive cycle of *Aedes aegypti*, 20E and EcR facilitate yolk protein production and fertility, inhibit the immune deficiency (IMD) pathway [20]. Host fitness is reduced under the pressure of pathogen invasion. For example, *B. bassiana* infection elicits *L. dispar* innate immune activation, but the digestive enzymes activity, feeding rate, and weight gain rate were inhibited [21]. To combat infection with *Metarhizium anisopliae*, *Locustana pardalina* experiences reduced food intake, substantial fat loss, and compromised dispersal and reproductive functions [22]. Artificial induction of melanization and humoral antibacterial activity leads to apoptosis of ovarian follicle epithelial cells in *Anopheles gambiae* [23]. Some studies have indicated that 20E signaling plays an important role in regulating insect growth, development, reproduction, and immunity, and also mediates trade-offs [24,25,26]. For example, C-Jun N-terminal kinase (JNK) modulates 20E levels to link reproduction and immunity in *Anopheles* mosquitoes [27]. *Calliphora vicina* larvae have robust innate immunity, yet increased 20E titers inhibit the expression of AMPs at pupation after *Escherichia coli* infection [28]. Phosphorylated and unphosphorylated FTZ-F1 affects the expression of *Bacillus thuringiensis* (Bt) toxin receptors via two distinct binding sites in *Plutella xylostella*, thereby conferring Bt resistance without growth penalty [29]. *B. thuringiensis* priming elevates *Spatzle* expression, which represses *Cyp315a1*, *Cyp314a1* and 20E synthesis, and thereby mediates the development–immunity trade-off in *Rhynchophorus ferrugineus* [30]. These findings confirm the coordinated regulation of growth and immunity by 20E signaling.

*Beauveria bassiana* infection triggers a robust innate immune response in the host, leading to delayed growth and eventual mortality [21]. The infection mechanisms of *B. bassiana* and the host immune responses have been well-characterized [31], but the role of 20E in antifungal immunity remains unclear. In this study, we first detected changes in endogenous 20E levels and analyzed the effects of 20E and JH on the growth and survival of the host during *B. bassiana* infection. Exogenous 20E treatment was conducted to evaluate its regulatory role in host antifungal immunity. Through proteomic analysis, we characterized the abundance of key proteins in the 20E signaling pathway during *B. bassiana* infection. RNAi-mediated knockdown was further used to investigate the role of Cyp306a1 in the antifungal defense of *L. dispar*.

## 2. Materials and Methods

### 2.1. Insects and Microorganisms

*L. dispar* eggs were collected from Chi Feng and Fu Xin in China. The eggs of moths were soaked in 10% formaldehyde solution (Chengshi, Chengdu, China) for 30 min, then washed with distilled water and dried. The *L. dispar* was placed in an artificial climate box (Dongtuo, Harbin, China) at 25 ± 1 °C, 14:10 (L:D). The artificial feed was purchased from the Chinese Academy of Forestry (Beijing, China), which mainly contained wheat germ, sugar, casein, etc. Healthy fifth instar of similar body size, one day post-molt, were selected as test insects.

*B. bassiana* were stored in the laboratory and cultured on Sabouraud Dextrose Agar with Yeast Extract (SDAY) (Glucose 40 g, yeast extract 10 g, peptone 10 g, and agar 20 g, dissolved in distilled water to 1 L.) for 10 days at 28 °C. Using 0.05% Tween-80 (Nachuan, Harbin, China) to prepare spore suspension and adjust the concentration to 1 × 10^6^/10^7^/10^8^ conidia/mL, each larva was injected with 2 μL. After 48 h post-injection (HPI), the larvae were collected and stored at −80 °C [21].

### 2.2. Determination of 20E Titer

After 48 HPI with *B. bassiana*, samples were collected. Each group consisted of ten larvae, and the experiment was repeated three times. Samples were weighed and pulverized in liquid nitrogen, then mixed with 1 mL methanol (Oceanpak, Tianjin, Chian). The homogenate was further processed with a tissue grinder, subjected to ultrasonic extraction in an ice-water bath for 30 min, and incubated overnight at 4 °C to complete the extraction. After centrifugation, the supernatant was collected, filtered through a 0.22 μm filter, and stored at 4 °C until analysis.

HPLC analysis was performed on a Waters 2695 system equipped with a UV detector set to 248 nm. Chromatographic separation was achieved on a Compass C18 (2) column (250 mm × 4.6 mm, 5 μm) maintained at 35 °C, using an isocratic mobile phase of methanol–water (50:50, *v*/*v*) delivered at a flow rate of 1.0 mL/min. The injection volume was 10 μL. Prior to injection, all samples were filtered through 0.22 μm membrane filters (Millipore, Burlington, MA, USA) and degassed by ultrasonication.

### 2.3. cDNA Synthesis

The total RNA of fifth instar larvae of *L. dispar* was extracted by the Trizol method, and then the RNA was reverse-transcribed into cDNA (YoungGen HP All-in-one qRT Master MixII kit, Kunming, China) according to the manufacturer’s instructions. The primers were designed using Primer Premier6, and the sequences of the primers are shown in Table 1.

### 2.4. Insect Treatments

**Figure 1:**
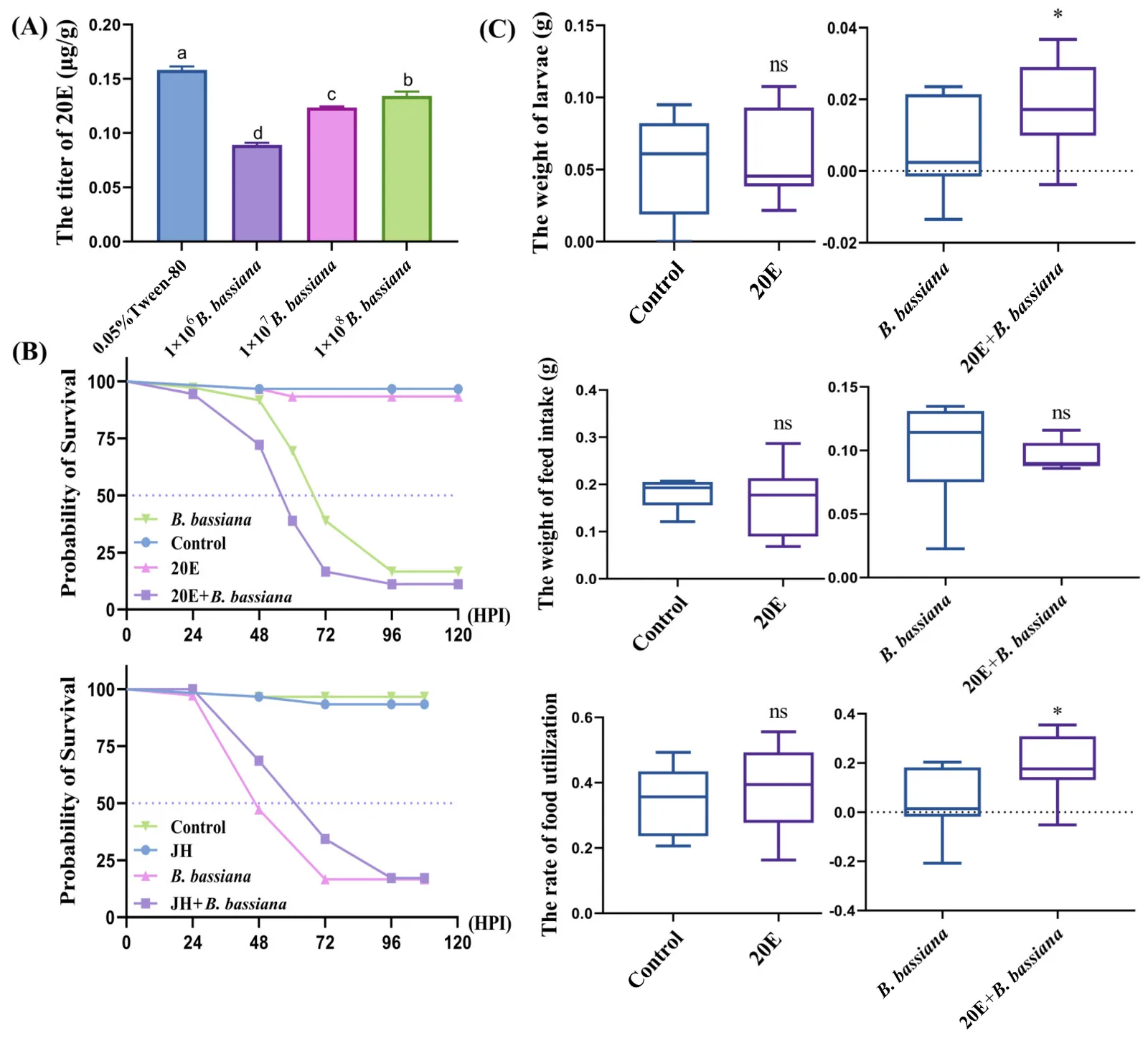
20E participates in the immune defense of *L. dispar* to *B. bassiana* infection. (**A**) 20E titer of *L. dispar* under *B. bassiana* infection. (**B**) Mortality of *L. dispar* after 20E/JH treatment. (**C**) Growth of *L. dispar* after 20E treatment. “*” and different letters represent statistically significantly differences. “ns” indicates no significant difference.

20E was purchased from Aladdin (Shanghai, China), and JH was purchased from KKL med (Guangzhou, China). 20E and JH were dissolved in anhydrous ethanol (Oceanpak, Tianjin, China), diluted to 15 ng/μL with double-distilled water, and injected as 2 μL per larva. The dose was selected based on Figure 1A. Each treatment group contained ten individuals, with three biological replicates. The mortality of *L. dispars* larvae was further detected. There were 40 larvae in each treatment, and each treatment was repeated three times.

W_G_ (g) = (W_2_ − W_1_)/D

F_C_ (g) = (W_f_ − Wi)/D

F_U_ = (W_2_ − W_1_)/[(W_f_ − W_i_) − F_O_]

W_G_: weight gain; W_1_: initial weight; W_2_: final weight; D: days; F_C_: food consumption; W_f_: final feed weight; W_i_: initial feed weight; F_O_: fecal output; F_U_: food utilization efficiency [32].

### 2.5. Cellular Immunity Determination

After injection, the hemolymph was collected on ice. The total hemocyte number was examined according to a previous study [33]. The encapsulation rate was further determined. Sephadex DEAE A-25 beads (Biosharp, Hefei, China), soaked in Congo red (Beyotime, Shanghai, China) solution in advance, were washed and added to the hemolymph at 25 °C for two hours. The gel beads were placed under a microscope (Leica DM2700 P, Leica Microsystems, Wetzlar, Germany) for the assay determining the encapsulation rate [34].

### 2.6. qRT-PCR

The expression of immune-related genes was investigated by qRT-PCR. qRT-PCR was conducted with 20 μL of PCR mixture (Vazyme, Nanjing, China) (SYBR Green Real-time PCR Master Mix 10 μL; nuclease-free water 6.5 μL; gene specific primers 1 μL, 0.5 mM; cDNA template 2.5 μL, 100 ng/μL). *Tubulin* and *EF-1α* were used as internal reference genes. The conditions for RT-qPCR reaction: 1 cycle at 95 °C for 30 s, 45 cycles at 95 °C for 12 s, 60 °C for 30 s, 72 °C for 40 s, and 82 °C for 1 s for plate reading. The expression levels were calculated with the 2^−△△Ct^ method [35].

### 2.7. Protein Preparation and Proteomic Analysis

Fat body tissues were dissected from *L. dispar* larvae at 48 HPI. Tissues from ten individuals were pooled as one biological replicate, and three independent replicates were prepared and stored at −80 °C. For protein extraction, samples were ground and sonicated in phenol extraction buffer (contain 1% protease inhibitor, Beyotime, Shanghai, China), mixed with an equal volume of Tris-saturated phenol, and then centrifuged. The resulting supernatant was precipitated with 0.1 M ammonium acetate (Guoyao, Shanghai, China) in methanol, washed sequentially with methanol (Thermo Fisher, Waltham, MA, USA) and acetone (Guoyao, Shanghai, China), and finally redissolved in 8 M urea (Solarbio, Beijing, China) [36].

Chromatographic separation was performed on a Vanquish Neo UHPLC system (Thermo Scientific, Waltham, MA, USA) operating at a nanoliter flow rate, equipped with a Thermo Scientific™ EASY-Spray™ column (2 μm C18, 150 μm × 15 cm; catalog no. ES906). The column oven was maintained at 55 °C, and the autosampler at 5 °C. Mobile phase A consisted of 0.1% formic acid in water, and mobile phase B of 0.1% formic acid (TGI, Shanghai, China) in 80% acetonitrile (Thermo Fisher, Waltham, MA, USA). The eluted peptides were subjected to data-independent acquisition (DIA) mass spectrometry on an Astral high-resolution mass spectrometer (Thermo Scientific, Waltham, MA, USA) [37]. Raw data were processed by database searching against the *L. dispar* genome for protein identification [38]. Differentially expressed proteins were identified using a threshold of *p* < 0.05 and a fold change >2 (up-regulated) or <0.5 (down-regulated). GO and KEGG enrichment analyses were carried out using clusterProfiler 4.0.

### 2.8. RNA Interference and Bioassay

Double-stranded RNA targeting Cyp306a1 (dsCyp306a1) and the control (dsGFP) were synthesized using the T7 RiboMAX Express RNAi System (Belong, Shanghai, China), verified by gel electrophoresis, and stored at −80 °C. Healthy 1-day-old fourth- and fifth-instar larvae of uniform size were selected for injection. A volume of 3.0 μL (2 μg/μL) of dsCyp306a1 or dsGFP was injected into the second abdominal intersegmental membrane using a microsyringe (Hamilton, Shanghai, China). Post-injection larval mortality was recorded daily. There were 40 larvae in each treatment, and each treatment was repeated three times.

### 2.9. Statistical Analysis

Data were analyzed using SPSS 22.0, and the quantitative results were processed using Bio-Rad CFX Maestro software 1.1. The statistical significance of differences was evaluated by Tukey’s test or one-way analysis of variance (ANOVA). Survival data were analyzed using Kaplan–Meier curves, and differences between groups were assessed by the log-rank test. Differences were considered statistically significant when *p* < 0.05. Bars sharing “*” or different letters represent statistically significantly differences. “ns” or the same letter indicates no significant difference.

## 3. Result

### 3.1. 20E Modulates Mortality and Development of L. dispar Against B. bassiana

*B. bassiana* was injected into the hemocoel of *L. dispar*, and the 20E titer was determined at 48 HPI. The results revealed that injection of *B. bassiana* significantly reduced the 20E titer in *L. dispar* compared to the Tween-80 group (*p* < 0.05) (Figure 1A). Based on previous studies [21,31], a suspension of 1 × 10^7^ conidia/mL of *B. bassiana* was chosen for subsequent treatment. 20E/JH + *B. bassiana* was injected into *L. dispar* to investigate larval survival. Exogenous 20E significantly shortened the median lethal time (LT_50_) compared to the *B. bassiana* group, while JH substantially prolonged it (*p* < 0.05) (Figure 1B). Exogenous 20E alone did not significantly affect weight gain, food intake, or food utilization efficiency in *L. dispar* compared to the control group (*p* > 0.05), but the 20E + *B. bassiana* group markedly improved weight gain and food utilization compared to the *B. bassiana* group (*p* < 0.05), though food intake remained unchanged (*p* > 0.05) (Figure 1C).

### 3.2. 20E Suppresses Immune Responses in L. dispar Against B. bassiana

**Figure 2:**
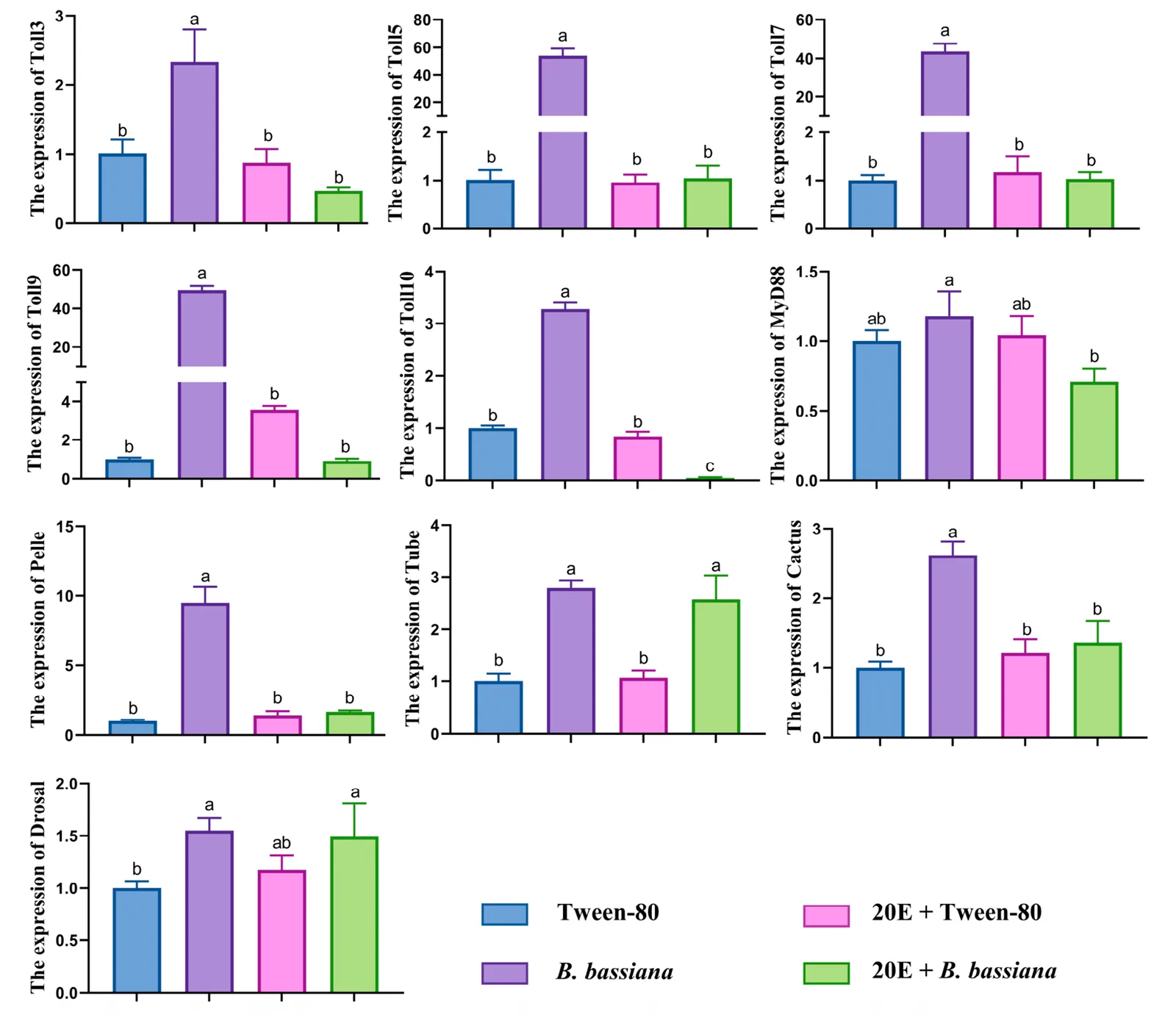
Effects of 20E on the Toll pathway in *L. dispar*. Different letters represent statistically significantly differences.

The Toll signaling pathway serves as the primary defense mechanism in insects against fungal infection [39]. The expression of genes related to the Toll signaling pathway in *L. dispar* were examined at 48 HPI. Except for *MyD88* (*p* > 0.05), invasion by *B. bassiana* activated the expression of *Toll3*, *Toll5*, *Toll7*, *Toll9*, *Toll10*, *Pelle*, *Tube*, *Cactus*, and *Drosal* in *L. dispar* compared to the Tween group (*p* < 0.05). Exogenous 20E treatment did not significantly alter the transcript abundance of Toll pathway-related genes compared to the Tween group (*p* > 0.05). Compared with the *B. bassiana* group, the 20E + *B. bassiana* group significantly decreased the expression of *Toll3*, *Toll5*, *Toll7*, *Toll9*, *Toll10*, *MyD88*, *Pelle*, and *Cactus* in *L. dispar* (*p* < 0.05), whereas the expression of *Tube* and *Dorsal* remained unchanged (*p* > 0.05) (Figure 2). In addition, quantitative analysis of AMP expression revealed that *Attacin2*, *Gloverin4*, and *Lysozyme4* expression were significantly decreased in the 20E + *B. bassiana* group compared to the *B. bassiana* group (*p* < 0.05) (Figure S1).

### 3.3. 20E Modulates Cellular Immunity in L. dispar Against B. bassiana

**Figure 3:**
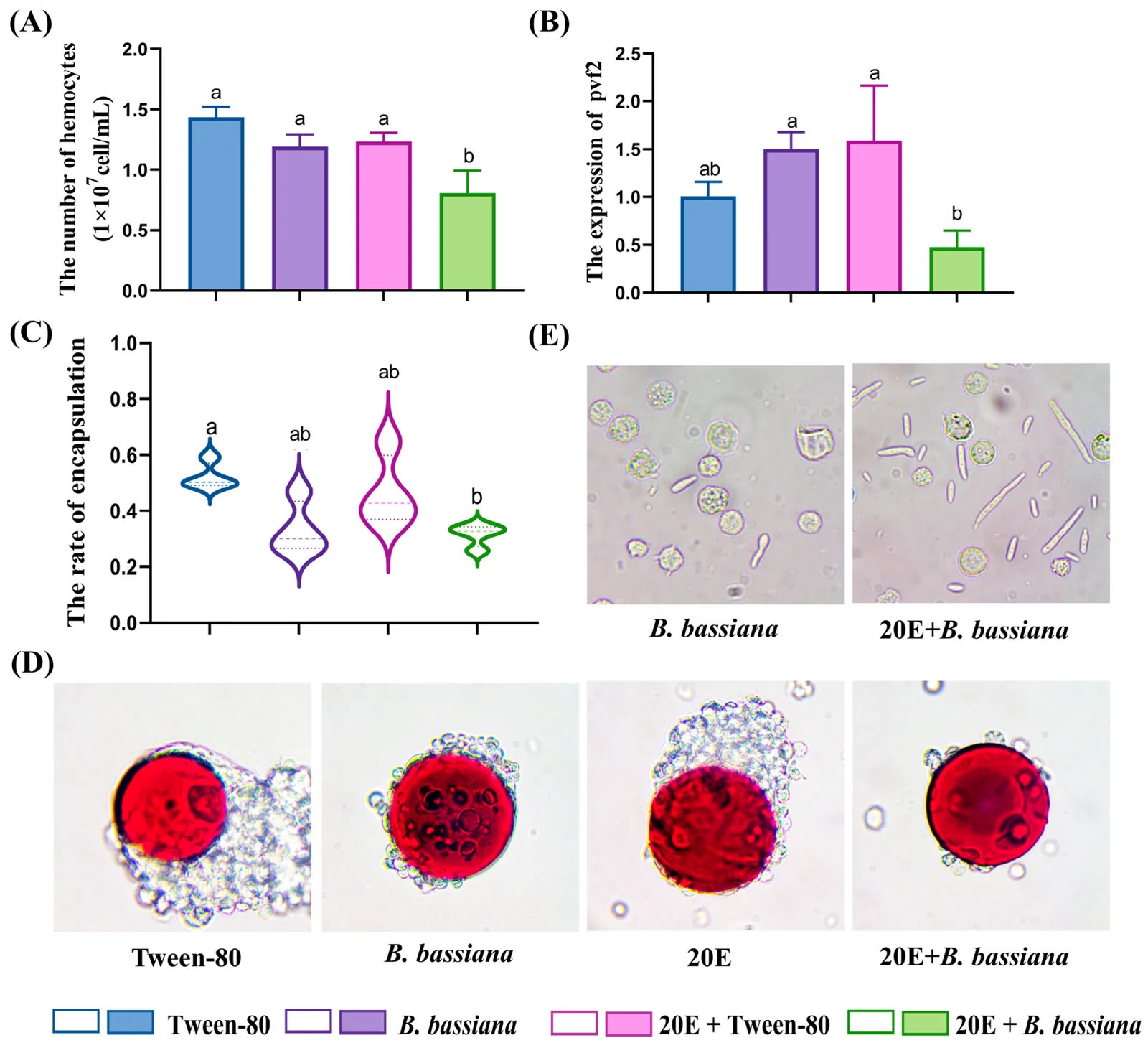
Effects of 20E on cellular immunity in *L. dispar*. (**A**) Total hemocyte counts. (**B**) Expression of *pvf2*. (**C**) Rate of hemocyte encapsulation. (**D**) Encapsulation of gel beads (red) by hemocytes. (**E**) Differences in hyphal body number. Different letters represent statistically significantly differences.

Hemocytes mediate cellular immunity, with immune function related to hemocyte numbers. Compared with the *B. bassiana* group, the 20E + *B. bassiana* group induced a significant decrease in total hemocyte counts at 48 HPI (*p* < 0.05) (Figure 3A). The expression of *Pvf2*, a gene involved in hemocyte proliferation was examined [40,41]. At 48 HPI, *Pvf2* expression was significantly decreased in the 20E + *B. bassiana* group compared to the *B. bassiana* group (*p* < 0.05) (Figure 3B). Compared with the Tween-80 group, the 20E + *B. bassiana* group significantly decreased the rate of encapsulation capability (*p* < 0.05), but the *B. bassiana* group and the 20E + *B. bassiana* group had no significant difference (*p* > 0.05) (Figure 3C,D). In addition, the 20E + *B. bassiana* group significantly increased hyphal body counts in hemolymph compared to the *B. bassiana* group (Figure 3E).

### 3.4. Proteomic Profiling of 20E Signaling Pathway in L. dispar Against B. bassiana Infection

**Figure 4:**
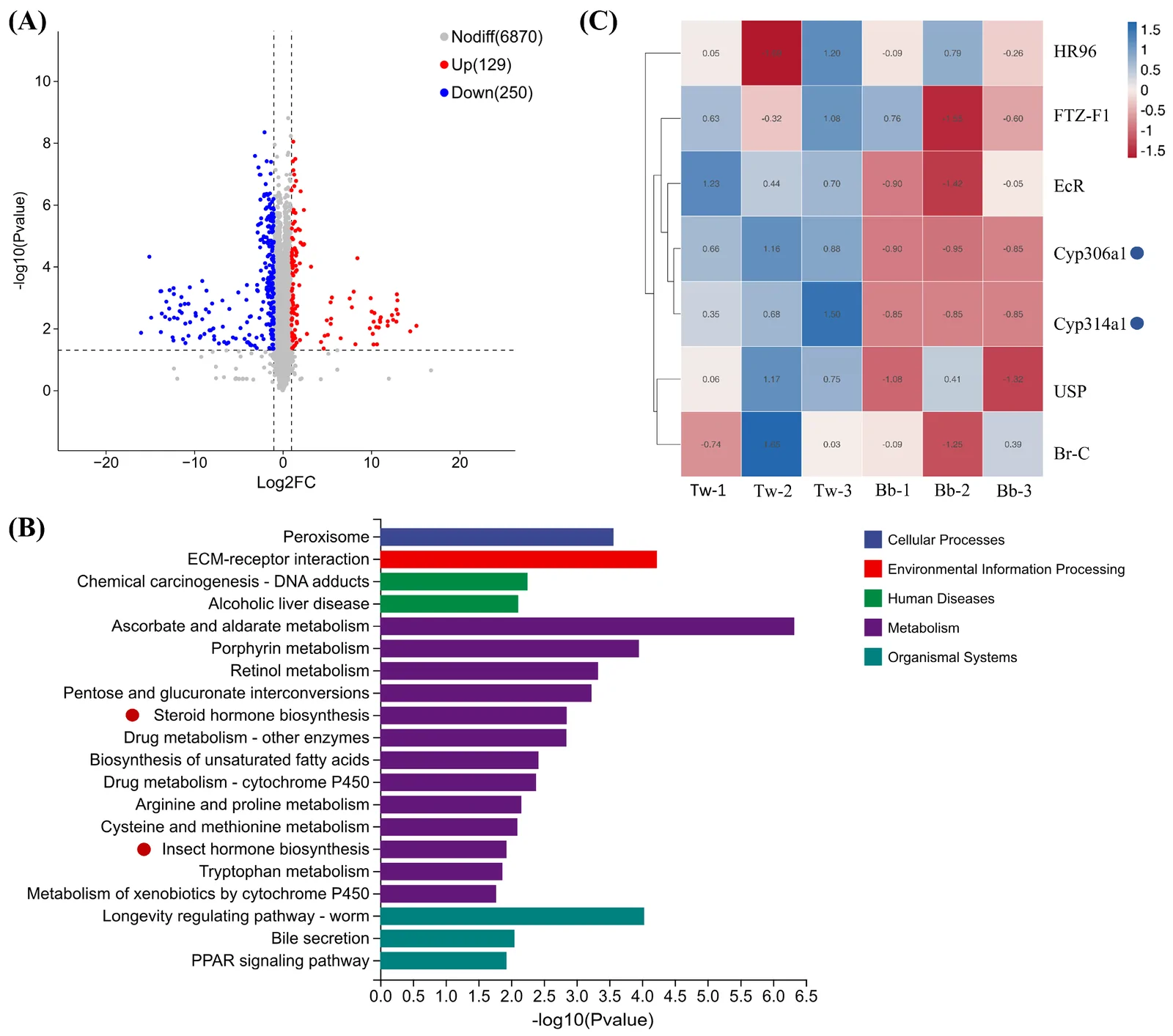
Proteomic analysis of 20E signaling pathway in *L. dispar* upon *B. bassiana* infection. (**A**) Volcano plot showing differentially expressed proteins. (**B**) KEGG pathway enrichment analysis. (**C**) Heatmap of 20E pathway-related protein abundance. (Tw denotes treatment with Tween-80, while Bb denotes treatment with *B. bassiana*.).

Proteomic profiling showed that *B. bassiana* infection up-regulated 129 proteins and down-regulated 250 proteins (Figure 4A). GO term enrichment was significantly associated with monooxygenase and oxidoreductase activities and heme and iron ion binding (Figure S2). KEGG enrichment analysis revealed that the steroid hormone biosynthesis, insect hormone biosynthesis pathway, and cytochrome P450-mediated drug and xenobiotic metabolism pathways were prominently enriched, suggesting that *B. bassiana* invasion may modulate hormone metabolic networks to trigger host immunity (Figure 4B). A heatmap plot showed the key proteins in the 20E signaling pathway. The results showed that Cyp306a1 and Cyp314a1 were significantly down-regulated in the *B. bassiana* group compared to the Tween-80 group (*p* < 0.05) (Figure 4C).

### 3.5. Cyp306a1 Regulates the Antifungal Immune Response of L. dispar

**Figure 5:**
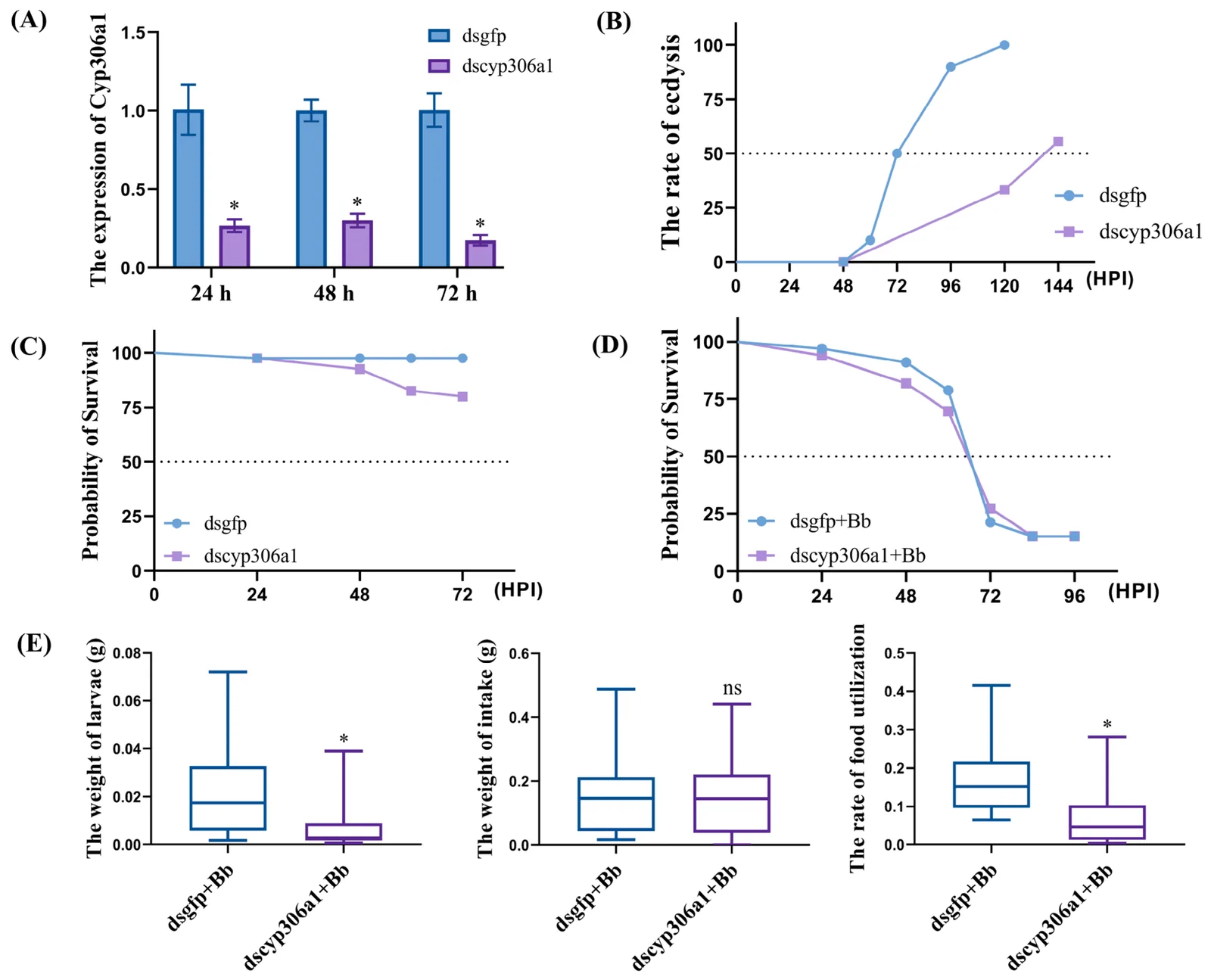
Effects of Cyp306a1 knockdown on *L. dispar*. (**A**) Knockdown efficiency of Cyp306a1. (**B**) Effects of Cyp306a1 knockdown on ecdysis rate. (**C**) Effects of Cyp306a1 knockdown on larval survival. (**D**) Effects of Cyp306a1 knockdown on antifungal capacity against *B. bassiana*. (**E**) Effects of Cyp306a1 knockdown on larval weight gain, food intake, and food utilization under *B. bassiana* infection. (Bb denotes treatment with *B. bassiana*.). “*” represents statistically significantly differences, “ns” indicates no significant difference.

To explore the roles of Cyp306a1 in antifungal immunity, we established an RNAi-mediated knockdown model in *L. dispar*. In the dsCyp306a1 group, the knockdown efficiency of Cyp306a1 was approximately 78–93% compared to the dsGFP group (*p* < 0.05) (Figure 5A). In *L. dispar*, knockdown of Cyp306a1 significantly delayed ecdysis, reduced 20E titers, and decreased survival compared to the dsGFP group (*p* < 0.05) (Figure 5B,C and Figure S3). However, there was no significant difference in survival between the dsCyp306a1 + *B. bassiana* group and the dsGFP + *B. bassiana* group (*p* > 0.05) (Figure 5D).

Compared with the dsGFP group, knockdown of Cyp306a1 had no significant effect on the growth, feeding, or food utilization efficiency of *L. dispar* (*p* > 0.05) (Figure S4). In the dsCyp306a1 + *B. bassiana* group, the weight and food utilization of *L. dispar* were significantly decreased (*p* < 0.05), but food intake remained unchanged compared to the dsGFP + *B. bassiana* group (*p* > 0.05) (Figure 5E).

**Figure 6:**
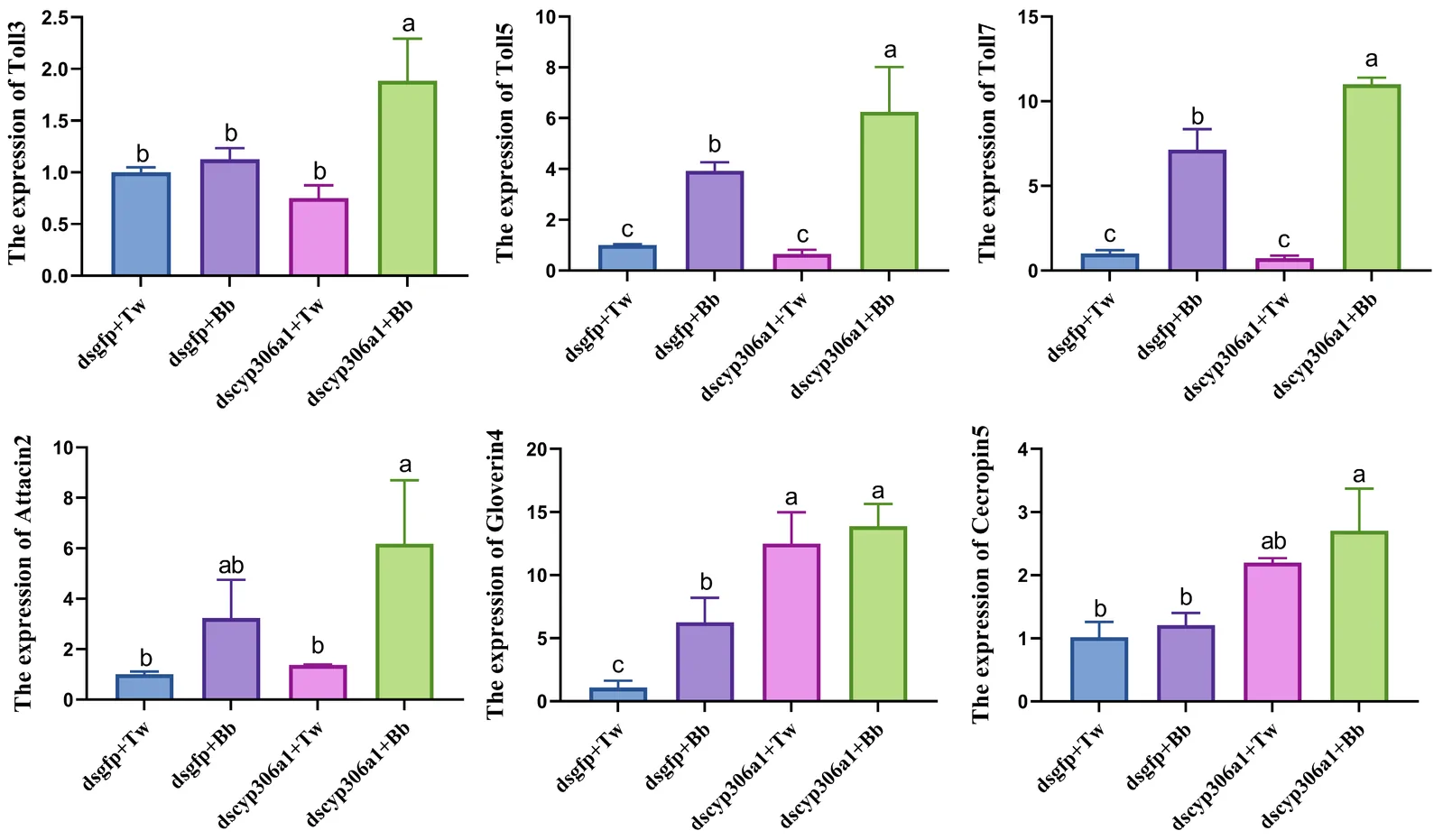
Effects of Cyp306a1 knockdown on Toll-related and AMP expression. (Tw denotes treatment with Tween-80, while Bb denotes treatment with *B. bassiana*.). Different letters represent statistically significantly differences.

To assess the role of Cyp306a1 in antifungal immunity, we measured the expression of Toll-related genes in *L. dispar*. Knockdown of Cyp306a1 significantly up-regulated *Toll3*, *Toll5*, *Toll7*, *Gloverin4*, and *Cecropin5* under *B. bassiana* infection (*p* < 0.05). By contrast, *Attacin2* expression was slightly increased, though the difference was not significant (*p* > 0.05) (Figure 6).

## 4. Discussion

Previous studies have extensively documented the immune responses and growth changes in *L. dispar* against *B. bassiana* [21,31]. However, the role of 20E in regulating antifungal immunity and growth of *L. dispar* remains unclear. In this study, we found that the 20E pathway serves as a key regulator of innate immune activation and mediates the trade-off between growth and immune defense in *L. dispar* upon fungal stress.

Some studies have shown that elevated 20E suppressed host innate immune responses. For example, a genome-wide microarray assay revealed that 20E inhibits the expression of *Moricin* and *Cecropin* in *B. mori* [14]. Exogenous 20E injection led to a significant decrease in *ctl13* and AMPs, whereas knockdown of EcR increased the expression of *Tcctl13* and AMPs in *Tribolium castaneum* [42]. In *Aedes aegypti*, knockdown of EcR enhanced the expression of IMD pathway components and improved tolerance to Gram-negative bacterial infection [20]. Consistent with previous findings, *B. bassiana* infection significantly decreased 20E titers compared to the control group. Exogenous 20E markedly accelerated the mortality of *L. dispar* during *B. bassiana* infection. Furthermore, the 20E + *B. bassiana* group markedly suppressed the expression of Toll-related genes, AMPs, and hemocyte numbers in *L. dispar* compared to the *B. bassiana* group. In summary, the decrease in 20E levels thus contributes to the activation of innate immune response and enhances the survival of *L. dispar* during infection of *B. bassiana*.

Some studies have demonstrated that elevated 20E levels can enhance insect immunity. For example, in *B. mori*, 20E (0.5 μg/larva; 2–4 μg/larva) increased AMPs, including Lebocin, boosting resistance to microbial infection [11,43]. In *L. migratoria*, injection of 20E (10 μg/larval) significantly increased the expression of PGRP-SA, AMPs, and lysozyme, thereby enhancing its immune function [13]. We attribute this inconsistency mainly to exogenous 20E dosage. In *B. mori*, 3 μg/larva 20E suppressed AMP expression, whereas 4–5 μg/larva markedly up-regulated it [14,44]. Similarly, we tested varying 20E concentrations in *L. dispar* (Figure S5); although a high dose reduced mortality, it far exceeded physiological 20E titers (30 ng/larva). Beyond dosage, the insect’s developmental and reproductive stages modulate 20E-mediated innate immunity. For example, mated females of *Gryllus texensis* showed higher resistance to *Serratia marcescens* than unmated ones [45], while mating reduced survival in *D. melanogaster* infected with *Pseudomonas aeruginosa* at 3 h post-mating [46]. PGRP and AMP transcripts were elevated in the fat body of wandering *H. armigera* larvae versus feeding ones. Furthermore, infection level also affects 20E titers. In Figure S6, a high-lethality dose (1 × 10^6^–10^8^ conidia/mL) of *B. bassiana* suppressed 20E levels in *L. dispar*, whereas a low-lethality dose (1 × 10^5^ conidia/mL) has no significant difference compared to the control group. Finally, it is worth noting that even at the same 20E dosage within the same host species, cellular and humoral immune responses can diverge. For example, in *H. armigera*, 500 ng/larva 20E inhibited plasmatocyte spreading but simultaneously enhanced the expression of PRRs and AMPs [12,15].

Upon pathogen invasion, hosts reallocate limited resources to balance growth and immune response [16]. For example, infection with *B. bassiana* triggered the innate immune response in *L. dispar*, accompanied by reduced activities of key digestive enzymes, feeding rates, and larvae weight gain [21]. In *Anopheles gambiae*, activation of melanization and humoral antibacterial responses induced apoptosis of ovarian follicle epithelial cells [23]. Consistent with previous findings, in *L. dispar*, *B. bassiana* invasion significantly reduced weight gain while enhancing the antifungal immunity through the reduction of 20E levels. Conversely, exogenous 20E not only inhibited larval resistance to *B. bassiana* but also promoted weight gain and food utilization, effectively offsetting the growth cost. These findings indicate that 20E acts as a key mediator of the growth–immunity trade-off during fungal infection.

The Halloween gene family (Cyp307a1, Cyp306a1, Cyp302a1, Cyp315a1 and Cyp314a1) encodes a group of enzymes essential for ecdysteroid synthesis [47,48]. In *Schistocerca gregaria*, *Frankliniella occidentalisand* and *Diaphorina citri*, the knockdown of Halloween genes results in a decrease in 20E titer, as well as delays the development of hosts [49,50,51]. Some studies have shown that the Halloween gene family is involved in the regulation of the host immune response. For example, co-exposure to triflumezopyrim and triadimefon significantly activated the expression of *Cyp306a1* in *Apis mellifera* L [52]. In response to *Bt* invasion, *R. ferrugineus* mediates immune activation trade-offs by suppressing the expression of *Cyp315a1* and *Cyp314a1* synthesis through Spatzle [30]. In this study, we analyzed the expression profiles of 20E signaling pathway in the fat body of *L. dispar*. The results revealed that *B. bassiana* invasion significantly decreased the expression of *Cyp306a1* and *Cyp314a1*. Knockdown of *Cyp306a1* significantly delayed molting compared to the dsGFP group, confirming that 20E synthesis was markedly inhibited. Meanwhile, in the dsCyp306a1 + *B. bassiana* group, the expression of antifungal immune-related genes was significantly increased, whereas weight gain and food utilization were significantly reduced in *L. dispar* compared to the dsGFP + *B. bassiana* group. These findings showed that down-regulation of 20E signaling allocates more resources toward innate immune activation in *L. dispar*. We have established that 20E titers and *Cyp306a1* expression modulate the antifungal immune response. However, our present findings do not allow us to definitively attribute the reduction of 20E levels in *L. dispar* larvae to Cyp306a1 activity. Therefore, future experiments will investigate whether restoring 20E levels in dsCyp306a1 larvae reverses the immune activation. In conclusion, *B. bassiana* infection reduces 20E levels in *L. dispar*, boosting antifungal immunity by reallocating resources from growth to defense. These findings enhance our knowledge of innate immunity in forest pests.

## Figures and Tables

**Table 1:** Primer sequences used in the experiment.

Usage of Primers	Gene	Primer Sequences
	dsGFP	5′-taatacgactcactatagggAGAAGAACTTTTCACTGG
dsRNA		3′-taatacgactcactatagggTGAACGGATCCATCTTC
	dsCyp306a1	5′-taatacgactcactatagggACCAACTGACGGCGAATGC
		3′-taatacgactcactatagggCGTGGCGGAGTAAGTGTAACT
Real-time PCR	qToll3	5′-ACGCCACGCTATATCATGGAATA
		3′-ACAGTCGCTTACGCACAACAT
	qToll5	5′-ATAGACGCCTTCAGAGACGAGAG
		3′-CGCTGTTCATGGTCCTTGGATC
	qToll7	5′-ACGACGCTTACAACGCAGTG
		3′-GTAGGTGGACAGTGGAATGACTC
	qToll9	5′-CCAGAATGGAGAGTCACAGCAT
		3′-CCTTATCGCAGGCAATGATTCAA
	qToll10	5′-CCGATCCGCTGAATACGTCTTC
		3′-TGACTCTTGACTCACATGCCTTC
	qMyD88	5′-AGCCGCTTGTCCTATCTTGCC
		3′-CCATTGAACATTCTCGCCACCTC
	qPelle	5′-GCGAATGGAAGCATCTCACAGT
		3′-ATGTTGTCGTGACGGTATTGGTT
	qTube	5′-TGGCACCATGTTGAACAATCTCT
		3′-ACGAGTACAGGAATGACACAAGT
	qCactus	5′-TCTGTTCTGTGTATTGCCAATGC
		3′-TGGTGCCTATAAGATCCGCCTAA
	qDrosal	5′-CGATTGCGACCTGCTTATGGAC
		3′-CGAGGACACAGTGGTGGTATTCT
	qGloverin4	5′-ACAGCAACAGGCTCAGGAGTG
		3′-TCACCAATCATGGCGGAACTCT
	qLys4	5′-TTGGCGAGCAGGCAAGAAG
		3′-ACCCGATAACAGATAGACGAAGG
	qAttacin2	5′-CGCAAGGTAGTCTCACACTCAAC
		3′-CCGAGGGCACTCAGCATATTCT
	qPv2f	5′-CGCAGACCTCACCATCGTTGA
		3′-GTGGCTGACTGTAAGGCAACTTG
	qCyp306a1	5′-TGGCAACCCTCCTCTTGAATTTG
		3′-CTAGCGGCTCACCGATGGAT
	qEF1-α	5′-TTTGCCTTCCTTGCGCTCAACA
		3′-TGTAAAGCAGCTGATCGTGGGT
	qTubulin	5′-AATGCAAGAAAGCCTTGCGCCT
		3′-ATGAAGGAGGTCGACGAGCAAA

Note: Lowercase letters indicate T7 polymerase promoter.

## Data Availability

The original contributions presented in this study are included in the article. Further inquiries can be directed to the corresponding authors.

## Supplementary Materials

The following supporting information can be downloaded at: https://www.mdpi.com/article/10.3390/insects17080839/s1, Figure S1: Effects of 20E on AMPs of *L. dispar*; Figure S2: GO enrichment analysis of the *L. dispar* proteome upon *B. bassiana* infection; Figure S3: Effects of Cyp306a1 knockdown on 20E titers in *L. dispar*; Figure S4: Effects of Cyp306a1 on growth of *L. dispar*; Figure S5: Dose-dependent effects of exogenous 20E on *L. dispar* resistance to *B. bassiana*; Figure S6: Effect of *B. bassiana* treatment at 1 × 10^5^ conidia/mL on 20E titers in *L. dispar*.
