# Supplementary material for: Reduced 20-Hydroxyecdysone Signaling Modulates Antifungal Immune Responses and Growth in Lymantria dispar Against Beauveria bassiana
Source: Insects. 2026 Aug 13;17(8):839. doi: 10.3390/insects17080839 (PMC13513474; doi:10.3390/insects17080839)
Supplement: Supplementary file 1 [file insects-17-00839-s001.zip › insects-4476345-supplementary.pdf]

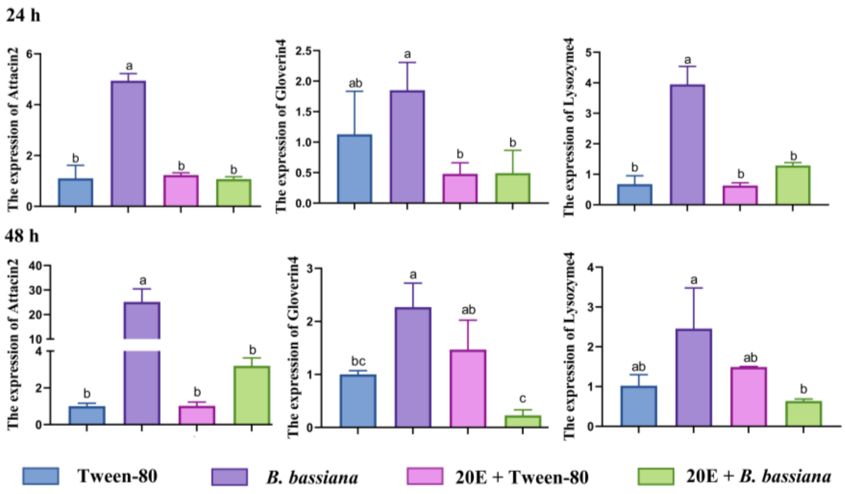

Figure S1 Effects of 20E on AMPs of *L. dispar*.

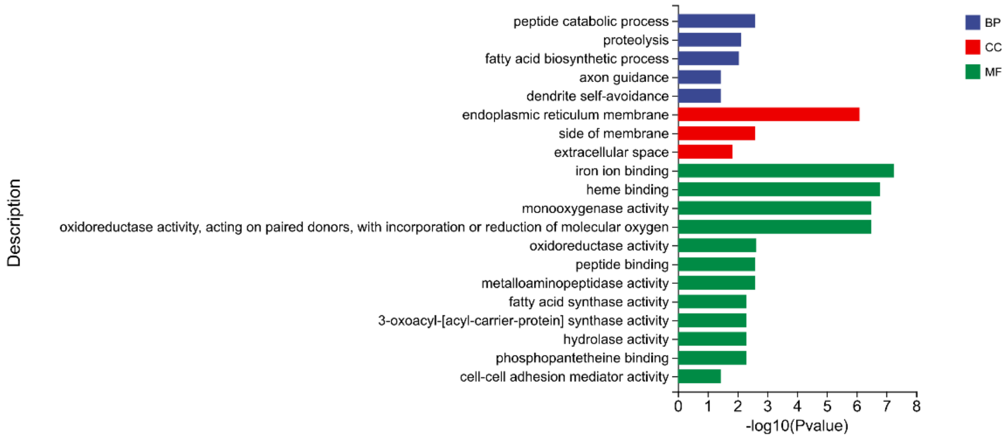

Figure S2 GO enrichment analysis of the *L. dispar* proteome upon *B. bassiana* infection.

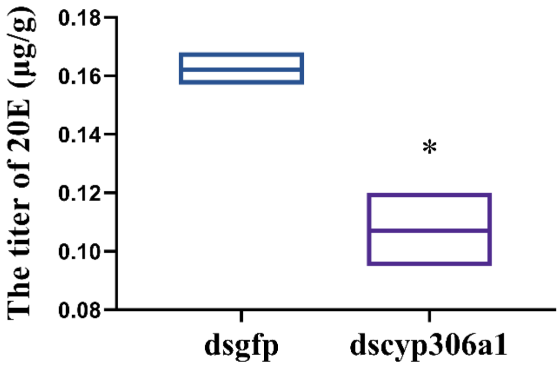

Figure S3 Effects of Cyp306a1 knockdown on 20E titers in *L. dispar*.

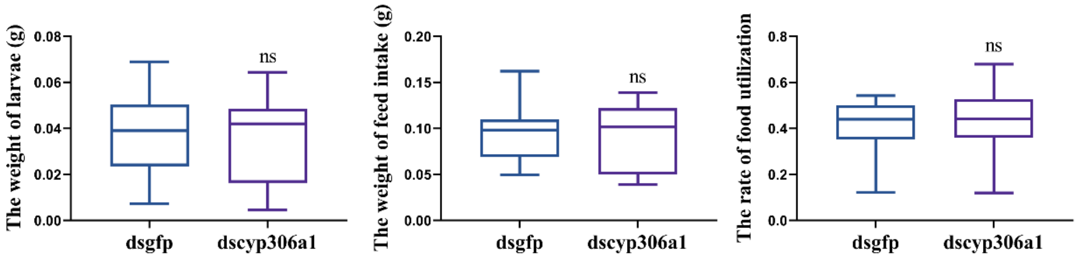

Figure S4 Effects of Cyp306a1 on growth of *L. dispar*.

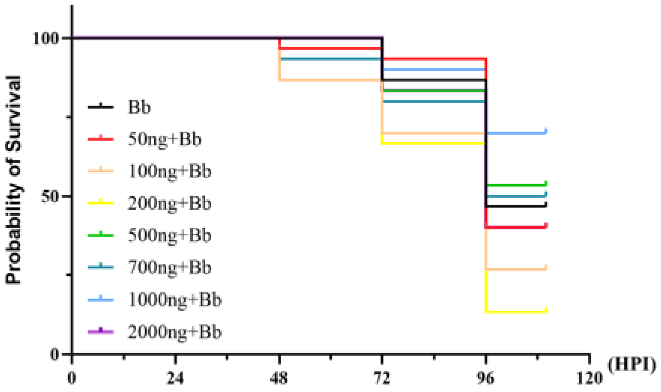

Figure S5 Dose-dependent effects of exogenous 20E on *L. dispar* resistance to *B. bassiana*.

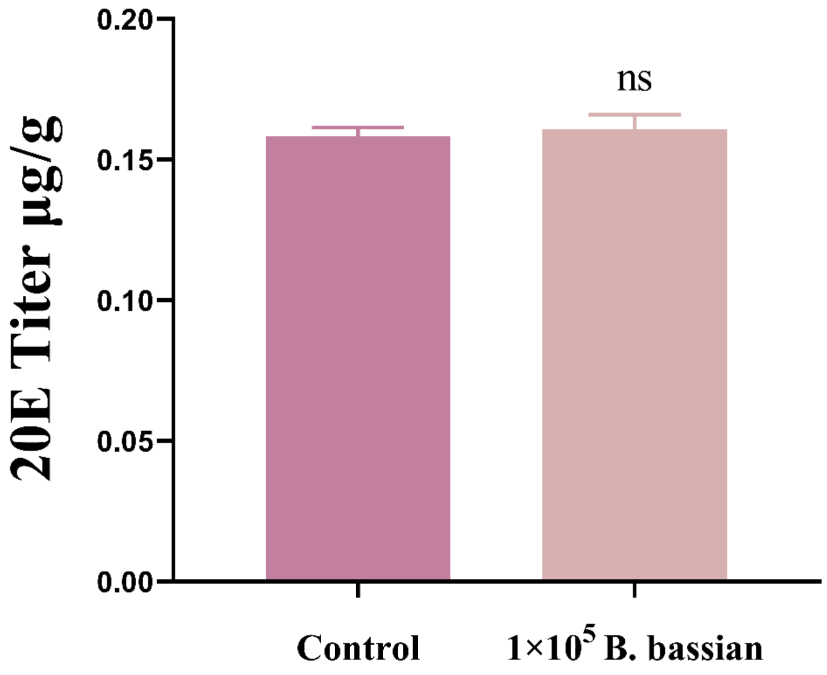

Figure S6 Effect of *B. bassiana* treatment at 1 × 10^5 conidia/mL on 20E titers in *L. dispar*
